# Medical Association of Georgia

**Published:** 1895-05

**Authors:** 


### Society Reports.

---

### MEDICAL ASSOCIATION OF GEORGIA.

Forty-sixth annual meeting, held in Savannah, April 17, 18 and 19, 1895.

### FIRST DAY—MORNING SESSION.

The association convened at the armory hall at 10 A. M., and was called to order by the president, Dr. Willis F. Westmoreland, of Atlanta.

Prayer was offered by the Rev. Dr. Dripps of the Independent Presbyterian church.

Addresses of welcome were delivered by Major Herman Meyers and Mr. J. F. B. Beckwith, and Dr. Frank M. Ridley, of LaGrange, responded on behalf of the association.

President Westmoreland then delivered his annual address in which he said the association was nearly half a century old, and only a few of the original members remained with us, and each year we noticed that a loved face was absent and learned that an honored friend had passed from our midst to return no more. Within the lifetime of the association the history of medicine had been revised and the better part of surgery written, and every subject pertaining to medicine had broadened out under the influence of broader ideas and the greater diffusion of knowledge; empiricism in medicine and surgery had almost disappeared, and that period when a great man's ideas, whether correct or not, was the standard he and his followers had for a guide, had passed away. Under modernizing influences, scientific methods of research were carried out by trained observers in well-equipped laboratories in every civilized country, and under these careful and patient investigations the misty surroundings of the causation of disease had begun to clear. Medicine in its wonderful progress had outgrown the individual capacity of any one and had resulted in an elaborate division of labor and the permanent and beneficial establishment of specialism; and able men by using their talent and genius along special lines, by their co-operative work had established the solid basis of the medicine and surgery of to-day. The specialist had to bear the brunt of a great deal of unjust criticism. In certain communities they had been censured as a class, when the blame was only merited by individuals. Unfortunately there were medical men who used the broad cloak of specialism to cover the most outrageous charlatanism, and in this respect the field of gynecology presented more rank growths than any other.

A few years before the organization of the association, in the upper portion of the State, there resided a country physician, a representative of that class of honest, earnest workers, who, from the viscissitudes of fortune, had plucked the courage to endure and the inspiration to strive; who, surrounded by pains, care and anxiety, was a man who ever proved a friend, comrade, counselor and guide. This physician was Dr. Crawford W. Long, of Jefferson, Georgia, who, by his discovery of anæsthesia and his successful use of ether in an operation on March 30, 1842, revolutionized surgery and started it upon its most progressive era.

On motion, the association adjourned to meet in the dancing hall of the DeSoto Hotel at 2:30 P. M.

FIRST DAY—AFTERNOON SESSION.

Dr. T. E. Mitchell, of Columbus, contributed a paper entitled "Traumatic Wounds of the Eye and Their Treatment," which was read by Dr. A. W. Calhoun, in the absence of the author.

For convenience of description, the author divided wounds of the eye into incised, punctured, lacerated and contused wounds. Another classification would be into aseptic and septic wounds. So likewise, we may have a simple compound or complicated wound. Simple, when the agent producing the wound and any other foreign substance carried by it into the rent is removed, and complicated when any foreign body, whether the missile inflicting the wound or material carried by it, remains in the injured member. The fundamental principles laid down for treatment of wounds in other parts of the body hold good here: (1) The arrest of hemorrhage. This will rarely be found troublesome. (2) Mitigate an existing shock. (3) Cleanse the wound of every vestige of foreign matter. (4) Render the wound absolutely aseptic by thorough irrigation with an antiseptic solution—corrosive sublimate 1-2000—5000. (5) Coaptation of the severed parts by sutures if necessary. (6) Antiseptic dressing, and (7) position and rest. The latter we accomplish with atropia or scopolamine, $\frac{2}{5}$ grain to 1 ounce of water, and a light pressure bandage to control the movement of the lids. The author then referred to wounds of individual parts of the eye. Wounds of the conjunctiva alone are generally caused by some corroding agent, as acids or alkalies, and offer a favorable or unfavorable prognosis depending entirely on the extent of corneal involvement. Superficial wounds of the cornea are among the most frequent of accidents, and are caused by the deposition in its structure of small pieces of steel, emery, grains of sand, cinders from a railroad locomotive, etc.

As to those cases in which enucleation is indicated, no definite rule can be formulated, since the intelligence, occupation and situation of the patient from the surgeon must be taken into consideration. Of course, the object of excision is to remove the peril of sympathetic inflammation. It is fortunate that sympathetic irritation usually precedes sympathetic inflammation, and serves as a signal for immediate enucleation. A wound involving the integrity of the zone of danger, or one in which a foreign body remains in the eye, is the most prolific cause of sympathetic disturbances. The consensus of opinion of the best authorities is in accord with the observations of Deutschmann that sympathetic ophthalmitis is of bacterial origin, the micro-organisms traveling by continuity of surface beneath the optic nerve sheath by way of the optic chiasm from the offending eye to the sympathizing member.

The prognosis of sympathetic inflammation is very grave, and the surgeon should not fail to so inform the patient, and if an attempt be made to save the offending eye, it should be done only after a thorough understanding of the risk assumed as to sympathetic involvement. As a substitute for excision, evisceration and optico-ciliary neurotomy has been advocated and practiced by some; but the consensus of professional opinion still adheres to enucleation as the safest line of procedure.

Dr. Louis H. Jones, of Atlanta, then read a paper entitled "Uranalysis."

The author said that the rapid strides made in physiological chemistry, and the more general use of the microscope, afforded us means for determining the composition of the urine with great accuracy and a minimum amount of labor. No greater mistake can be made than to assume that the condition of the urine is an index to the state of the kidneys alone. Disorders of the brain, the liver, the heart, the stomach, alike tell their tale in altered conditions of the urine. The author discussed only the most reliable and simple methods of detecting such changes and pointed to a few of the sources of error present in some of the methods in common use. There are, in making an examination of the urine, four points usually determined, namely: reaction, specific gravity, and the presence or absence of albumin and sugar. To these should always be added a knowledge of the amount passed in 24 hours and the quantity of urea present, and where there is reason to suspect disease, or where the chemical analysis indicates it, the microscope should always complete the work. The reaction can be readily determined by means of litmus paper, the only point worthy of consideration being, in the event of an alkaline reaction, to know whether this represents the state of the blood, or whether it is due to fermentive changes by means of which the urea has been split up with the formation of ammonium carbonate. Should this be the cause of an alkaline reaction, the litmus paper will regain its red color upon drying and warming. The urinometers in common use the author had found to be exceedingly unreliable, different instruments giving, with the same specimen, variations ranging from 2 to 6 points. The chemical tests generally made of the urine are a reproach alike to the chemist and the physician. What is to be desired are tests which are reliable under all ordinary conditions, which can be made without the consumption of too much time and which require a minimum amount of skill for their proper performance. For the detection of albumin and for routine work, the essayist is partial to the ferrocyanide test to the exclusion of all others. It has to commend it both simplicity and reliability. It is made by adding, say to eight grams of urine, about six grams of a solution of potassium ferrocyanide (strength 1-20), and then adding one gram of acetic acid, when, if albumin be present, a distinct cloudiness will appear throughout the mixture.

The author called attention to the fact that often much unnecessary confusion is brought about by speaking of the percentage of albumin found in a given specimen of urine. As a matter of fact, the actual weight percentage of albumin present in urine never exceeds 3 to 4 per cent., and rarely exceeds one per cent, and yet it is common to hear of urines containing from 50 to 75 per cent. of albumin, reference being had to the bulk of the precipitate formed, usually with heat and nitric acid. This must necessarily depend much upon the time allowed for settling and is wholy inaccurate and unscientific. The author then referred to the various methods for detecting sugar in the urine. In closing he calls attention to the centrifugal method of obtaining urinary sediment for microscopic examination. By means of a small electric motor, carrying two arms with tubes attached, and which can be run by a small battery, the urinary sediment may be obtained in a few minutes.

Dr. A. W. Calhoun, of Atlanta, read a paper entitled "The Management of Hemorrhage after Tonsil Operations."

The frequency of alarmingly profuse hemorrhage after tonsil operations, with an occasional fatal result, makes this a subject of exceeding interest to the surgeon. Such accidents should not, however, stand as obstacles in the way of performing these operations, for the necessity for operation is both frequent and urgent. Especially in children is the enlarged or hypertrophied tonsil very often met with, and the evil effects of the disease are so apparent to the medical and even non-medical observer, that it often calls for prompt action on the part of the physician. The ordinary hemorrhage following the operation is of but small consequence, as with a little patience and care the blood soon ceases to flow of its own accord; or, if need be, a gargle of cold salt water suffices to arrest it in the course of a few minutes. But when there flows from the cut surfaces a steady stream of blood continuing for hours, then it is that the tact and nerve and skill of the surgeon display themselves to the greatest advantage. With an experience of nearly 4,000 tonsil operations, the author had seen but few cases of alarming hemorrhage in children. Young children are not very liable to dangerous hemorrhage, but it is in the adult that the great danger lies. The actual cautery applied directly to the bleeding surface, the author could not suggest, but he could recommend pressure directly applied to the wound, which is the most satisfactory of all means. Pass the forefinger, the end of which is covered with a piece of moistened sponge or absorbent cotton, into the mouth and carefuly cover the cut surface, and with the wound between the fore-finger of the one hand and the palm of the other hand placed externally, exercise gentle but steady pressure. The hemorrhage ceases immediately, but recurs upon the removal of the pressure. It is now a matter of courage and confidence on the part of the patient and of physical endurance on the part of the doctor. In most cases pressure continued for ten minutes to one or two hours suffices to permanently arrest the hemorrhage, but in rare instances it must be continued through twelve to twenty-four hours. In these last cases assistance must be called in, so that the persons exercising the pressure can be rested at intervals of half an hour. Dr. Calhoun had never known this mode of checking hemorrhage to fail, and he recommends it to any practitioner having such a case.

Dr. R. J. Nunn, of Savannah, read a paper with the following caption: "Something New in the Treatment of Women after Confinement."

It is well-known that women living in a condition most nearly approaching a state of nature do not require the "days of lying up" after confinement, nor do they keep them, as do the more civilized races, or the cultivated classes of society. The subject mentioned in the paper was a multipara who, after the birth of her first child, had suffered with prolapsus accompanied with cysto and rectocele, continual backache; was generally puny, miserable and run down, and was at best a small weakly woman, but withal a most determined character, as will be seen later. One Sunday morning the patient was delivered and immediately expressed her determination of going to her work on the next day. No persuasion could change her resolution.

Being a widow, she declared it to be a question of bread for herself and her children. Under these circumstances it was determined to adopt the following method of treatment: Twice daily the patient was to use a douche of a warm solution of boracic acid, immediately after which she was to report at Dr. Nunn's office. Here the uterus would be thoroughly cleaned out with aseptic water, and swabbed out with campenodine (camphor iodine and carbolic acid), the faradic current used with a vaginal electrode, a tampon applied, and an abdominal bandage was to be constantly worn. The treatment was carried out for about two weeks, after which it was gradually discontinued, and in about a month the patient was discharged apparently cured, not having had a single untoward symptom and declaring that her recovery had been better than after her first confinement, when she kept her bed ten days. Since this course of treatment, which is now several months ago, she has been continually at her work, not having missed a day. She enjoys better health than for years before and considers herself cured of her prolapsus.

As a guide to any one desiring to make further trial of this method of treatment, the author recapitulated the principles upon which it is founded: (1) Thorough antisepsis; (2) effective internal and external support; (3) local stimulation; to hasten involution of the uterus and the return of other overstrained parts to their normal condition; and, (4) where necessary, the use of such appropriate general medication as may be indicated.

### SECOND DAY—MORNING SESSION.

The first paper read at this session was by Dr. Dunbar Roy, of Atlanta, entitled "The Proper Care of School Children's Eyes—The Necessity."

The author summed up the main points of his paper, as follows: (1) That the hygiene of school children's eyes should receive as much attention as that of any other portion of the body.

(2) That in many cases where there is seeming physical ailment and mental inaptitude such as headaches, dull feelings, inattention, and loss of the power of concentration, the cause can be found in some ocular defect.

(3) That as myopia or nearsightedness increases as the pupil advances to higher grades, such treatment should be instituted as will arrest the progress of that pathological condition.

(4) That teachers should be instructed by competent opthalmologists as to the hygiene of the eyes and the various symptoms which indicate some ocular defect.

(5) That every child should have his or her eyes examined before entering school, and if necessary, a certificate should be given to that effect.

Dr. Frank M. Ridley, of LaGrange, read a paper entitled, "A Duty of the State Medical Association." His remarks referred principally to the State asylum, in which he said there had been dissatisfaction expressed as to the existing conditions and disappointment as to practical results. Most physicians were satisfied that inadequate legislative appropriations to a great extent lay at the bottom of these evils. According to the latest reports, there are now about 1,800 inmates in the asylum. So densely packed are these unfotunates that fresh applicants are frequently of necessity refused admission to the place on the ground that it would be dangerous to allow the number of patients to increase. As a consequence, in a deplorable number of instances, filthy county jails, swarming with vermin and poorly furnished with beds and covering, are converted into temporary sanitariums for the treatment of the insane. Dr. Ridley urges that another scheme of investigation should be inaugurated, namely: Let the governor appoint a committee composed of seven of the ablest physicians to visit the asylum once a year, and not at stated times. Let them be clothed with full powers to make a searching, sifting examination of every department from the kitchen to the superintendent's office. Submit to his excellency a carefully prepared report to be transmitted by him to the legislative body, suggesting such improvements in sanitation and general conditions as may seem to them to be urgent and feasible. Let them urge greater attention to the moral treatment of the inmates, as may be suggested by expert study and investigation of the best systems of Europe and

America. This will require some outlay of money. Money thus spent is well-spent, and should be met by the funds of the State treasury.

Dr. Ridley then referred to the inadequacy of the medical staff to perform the vast amount of professional labor assigned it. For the medical treatment of the 1,800 inmates referred to, suffering a score or more of nervous ailments, there are not exceeding five on the medical staff, a ratio of 600 patients to one physician, proportionately a greater charge on the physicians of the Georgia Insane Asylum than of any other similar institution, not only of the United States, but of the world. Dr. Ridley, in closing, urged the association to memorialize the governor to co-operate with a committee from the association looking to the remediation of these conditions.

After a free discussion of this paper, the president appointed a committee of five to act upon the recommendations and suggestions contained therein.

Dr. J. M. Hull, of Augusta, contributed a paper entitled, "Graves' Disease, with Cases."

Dr. W. S. Armstrong, of Atlanta, followed with a paper entitled, "Report of Several Interesting Operations."

The first case was one of gastrostomy for impermeable stricture of the œsophagus. The patient was 30 years of age, and a printer by trade.

Case second was an interesting one on account of the size of the stone removed from the bladder. In this case a suprapubic operation was performed. The stone was of enormous size, elliptical in shape, flattened on two sides, and weighed at the time of removal 13¾ ounces. Its greatest circumference was 12 inches, being larger by ⅓ than the stone removed by Dr. White, of Philadelphia, which has heretofore been regarded to be the largest stone successfully removed from a living person in the United States, without slough and without damaging the tissues. These stones compare as follows:

White's—weight, 9½ ounces; Circumference, 8½ inches. Armstrong's—weight, 13¾ ounces; Circumference, 12 inches, and 9¼ inches at right angles.

Patient recovered and now works on a farm.

Case third was one of operation for gunshot wound of the abdomen.

Dr. F.W. McRae, of Atlanta, read a paper entitled, "Appendicitis—A Brief Review of My Personal Experience." In the paper he said that in every case where there is a sudden attack of abdominal pain, especially if there be nausea and fever, appendicitis should be suspected and carefully sought for. In many cases it can be excluded, but it is always important to bear in mind the possibility of appendicitis and make a diagnosis at the earliest possible moment. Even in the beginning there are signs which point to appendicitis with more or less certainty. One of the first signs that may be observed is limited movement of the abdominal muscles of the right side during respiration. The four cardinal symptoms as laid down by Dr. Murphy are sudden attack of pain over the appendix; always nausea, frequently vomiting; elevation of temperature. Exaggerated local tenderness in various positions occupied by the appendix should always be borne in mind. Occasionally the pain and tenderness become localized on the opposite side of the abdomen or in the epigastric region. In some cases it is referred especially to the bladder, and the only symptom complained of is pain in the bladder, and either difficulty in emptying it or frequent urination. In two of the cases which the author had operated upon this symptom had been especially well-marked. In the typical cases of appendicitis a positive diagnosis is often difficult, sometimes impossible, prior to opening the abdominal cavity. Except in the fulminant cases, the treatment should be medical in the beginning. The bowels should be thoroughly purged, the diet regulated, counter-irritation or cold applied over the seat of pain and tenderness, and the progress of the attack watched carefully. Where the symptoms do not subside after careful purging, and local treatment is indicated, the case should be considered from a surgical standpoint, and where the pain and tenderness are increasing, the general symptoms becoming more and more marked, an operation should be done without delay, where favorable conditions and a good surgeon are at hand. Since the last meeting, the author had seen in his own practice and in consultation, nine patients suffering with appendicitis, and two of them having had each two attacks. He had also seen one patient, referred to him by the patient's physician, who had treated him through several attacks of recurrent appendicitis in the intervals between the attacks Of these ten patients he had operated on four, and assisted Dr. Nicolson in one, making five cases that had come to operation. Of the five cases operated upon, three of his, and the one operated upon by Dr. Nicolson, recovered. One of the cases operated upon by himself was an old man, who died nine weeks after the operation from exhaustion and paresis of the bowels.

Dr. McRae then gave histories of these cases.

SECOND DAY—AFTERNOON SESSION.

Dr. Wm. O'Daniel, of Bullards, read a paper entitled, "The Hygiene of Prison Camps in Georgia."

Dr. Virgil O. Hardon, of Atlanta, read a paper on "Laparotomy During Pregnancy."

It is comparatively seldom that the surgeon is called upon to open the abdomen of a woman who is normally pregnant. When conditions present themselves which would justify such a procedure in the non-pregnant woman, the existence of pregnancy offers a complication which leads us to consider seriously whether the interests of the patient would not be better served by postponing the operation until after confinement. There is always present a fear of interruption of the process of gestation involving a sacrifice of the life of the child in an effort to save that of the mother. Many cases therefore which would otherwise be legitimate subjects for abdominal section can no longer be so regarded when two lives are at stake instead of one. Urgent symptoms threatening one or the other of these lives constitute the sole indication for surgical interference under such circumstances.

The altered conditions imposed upon the pelvic organs by pregnancy give a new and entirely different aspect to many diseases. The physiological engorgement and the rapid structural changes which accompany pregnancy give to such diseases a different clinical history, and must be taken into consideration in discussing the prognosis and the treatment. The rapid development of tissue, most notable in the womb, but present also in other organs, extends to the new growths in these organs and causes rapidity of development of pathological processes proportionate to the rapid development of normal tissue. This fact must always be borne in mind as a factor in the prognosis. Another question to be seriously considered in such cases is the effect which the diseased condition will have upon the development of the fetus in utero. Still another point to be considered is the effect which the operation itself will have upon the continuance of pregnancy. Experience has proven that operations for the removal of the ovaries and tubes and even pedunculated subperitoneal fibromas are not incompatible with the continuance of pregnancy. It has been shown that such operations not only do not produce miscarriage, but that they have no effect upon the subsequent development of the fetus. Probably the most frequent pathological condition that we are called upon to consider in this connection is that of fibroma of the utero.

The technique of laparotomy upon the pregnant woman differs in no material respect from that of ordinary operation.

Dr. Hardon then reported two interesting cases in which he had opened the abdomen during pregnancy.

Dr. James H. Shorter, of Macon, read a paper on "Preventable Blindness, Especially as Related to Sympathetic Ophthalmia, with Reports of Some Typical Cases."

The author stated that most writers and all text-books divide sympathetic affections of the eye into two classes—sympathetic irritation and sympathetic inflammation. Some observers hold that the conditions are two distinct phenomena; that sympathetic irritation is a functional trouble, a reflex phenomenon, brought about through the agency of the ciliary nerves, while sympathetic inflammation is an organic affection, a genuine irido-cyclitis from the start, set up probably by a transference of sepsis by way of the optic nerves. The fact that in sympathetic irritation, so-called, the removal of the injured eye procures immediate and permanent relief from all symptoms of annoyance in the fellow-eye, whereas in genuine sympathetic inflammation such operation exerts but little, if any, influence in arresting the course of the disease, adds much probability to the truth of this theory. The author then dwelt upon the symptoms of sympathetic ophthalmia.

The treatment of sympathetic inflammation is chiefly antiphlogistic, rest in bed in a dark room, hot applications, leeches, atropine, etc., but any and all treatment promises at best but little when once the affection has declared itself. Prophylactic treatment, on the other hand, is always infallible, and may be summed up in a few words—remove the injured eye, and to this may be added the further advice, do this in good, time at once, if possible. The essayist briefly alluded to one case, which, from the sudden onset of the sympathetic inflammation, its destructive course in spite of early recognition of the condition and intelligent, vigorous treatment resorted to, was a typical example, and illustrated also the probable futility of all remedial efforts. The author laid down as an invariable rule that every eye made totally or partially blind by a penetrating wound, and especially if the globe remained tender to touch, should be removed without delay. Even if there be some reasonable amount of sight still left in the wounded eye, this is not a contraindication for the operation, for it may be set down as almost a certainty that a severely injured eye in which attacks of inflammation recur will be sooner or later lost.

Dr. Wm. Perrin Nicolson, of Atlanta, made some remarks on "Ligation of the External Carotid Artery as a Preliminary to, and Co-incident with, Operations upon the Jaws." He stated that in the history of ligation of arteries the external carotid artery had stood for a long time as a vessel that could not be interfered with, but that Dr. Wyeth had concluded from his investigations on the subject that ligation of this vessel was feasible. Dr. Wyeth's work on surgery contains a full account and description of the normal anatomy of the vessel, and its several variations, and the steps necessary to close it. The important feature about operations upon the external carotid is that in any trouble involving the external structures, in the face, about the mouth, etc., we can entirely cut off the circulation without interfering with the circulation in the brain, which has always caused a large mortality in the ligation of the common carotid. Some years ago, in an operation for removal of the upper jaw, Dr. Nicolson concluded to try the ligation of this vessel with a view to lessening hemorrhages at the time of the operation, and more especially in lessening the danger of return of the disease by a process of starvation, cutting off the blood supply from the seat of the growth. Since that time he had made the operation four times, always co-incident with operations for the removal of the jaw, and more especially in connection with operations for malignant disease. He has had no untoward circumstances connected with the four operations which he has made, and he feels certain that there is a wide field of usefulness ahead of this operation when it becomes more generally adopted, because the vessel is one which is easily reached and there can be no danger to the patient if the operation is properly performed.

"Scopolamine as a Mydriatic in Comparison with Atropine and Homatropine—A Postscript to a Previous Paper." This was the title of a paper contributed by Dr. Arthur G. Hobbs, of Atlanta.

Scopolamine hydrobromate has been demonstrated to have some superior qualities as a mydriatic: (1) In the small dose necessary, (2) in the few minutes required to produce complete paralysis of accommodation, and (3) in the comparative short time of its continuance. The author had used it in his refraction room almost exclusively in cases where atropine was not particularly indicated. The strength of the solutions has varied from one-half to one-eighth per cent., and in the latter weak solution he finds that a complete mydriasis is accomplished as effectually and it seems as rapidly, as when the forty times stronger solution is instilled. While he has, as a rule, used a one-fifth to a one-tenth per cent. strength, he does not believe that in these stronger solutions the result has been either more certain or has been accomplished in a shorter time. By all the comparative tests, the weaker solutions have given quite as good results as the stronger, and in both the results have appeared to be the same as when atropine or homatropine has been depended upon as the mydriatic.

In the few cases of glaucoma in which he could risk so powerful a mydriatic, no increase of tension was manifested. He does not doubt that we will find the results of the 1-160 per cent. solution, which he is now testing, will prove effectual. If so, when it is remembered that we use only from four to six drops from the pipette, the great potency of this drug is almost without a parallel. The author is using it in iritis when atropine does not promptly dilate, also in some forms of keratitis with increased confidence.

Dr. J. C. Le Hardy, of Savannah, read a paper entitled, "The Use of the Galvanic Current in Office Practice," in which he reported several interesting cases treated by this current with gratifying results.

"Report of a Part of My Surgical Work for the Six Months ending April 15th, with Remarks." This paper was read by Dr. J. B. S. Holmes, of Atlanta. The author reports fifty-one cases.

In no case had an ovariotomy been performed simply for the relief of nervous or reflex symptoms. He had operated only for disease—conditions that he knew could only be relieved by surgical procedure, and careful examination, post-operative, had confirmed his opinion in every instance. While he believes in conservative measures, the most radical are often the most conservative. He is ready to admit that the abdominal cavity had often been ruthlessly invaded and organs removed that should not have been touched. They had been removed for nervous symptoms due entirely to other causes, and for which they were in nowise responsible. On the other hand, tinkering gynecology was responsible for many diseased tubes and ovaries that could only be cured by removal. The introduction of various caustic remedies into the uterus caused destruction and sloughing of the mucous membrane, with a cervical canal in many cases previously constricted, and so rendered by the application that the decomposing masses could not pass out. What follows? A septic endometritis which soon extends to the tubes and ovaries, producing trouble that nothing but the knife will relieve. This must be resorted to or the poor woman is frequently doomed to permanent invalidism, a condition sometimes worse than death. Many of the cases that the surgeon is censured for using the knife upon are caused by such treatment. He does not condemn all local treatment; when properly directed, he believes there are many conditions where it is not only admissible but demanded. He does not wish to be understood as advocating the removal of every diseased ovary. Far from it. He declines operation in many cases where the patient is anxious for it. He tries to be conscientious and operates only where the disease is so advanced that it has or is likely to render the woman an invalid, and is incurable by any other means.

The author's experience with catgut as sutures or ligatures has been unsatisfactory. He never uses it except when he does a trachelorrhaphy and perineorrhaphy, at the same sitting. It is hard to sterilize, is soft, slips easy, is hard to tie with any assurance of safety, and is absorbed too quickly.

While he had always deprecated the use of opium in abdominal work, he had used it with great care and caution. Increasing experience convinces him that those cases in which it is not given at all do much better and their convalescence is more satisfactory in every respect. Where the pain is so intense and threatens to exhaust the patient from continued suffering, he thinks it advisable to use opium moderately and with great care.

At the conclusion of the paper, Dr. Holmes exhibited a few specimens removed from women who had been the victims from one to three years of the so-called conservative treatment with iodine, iodized phenol, nitrate of silver, nitric acid, carbolic acid, and other similar caustics.

Dr. T. M. Greenwood, of Mineral Bluff, read a paper on "Obstetrics as Practiced in the Mountains of North Georgia."

The author said that a prominent laparotomist of the South had stated that healthy mothers were generally playing out, but thinks his observation was largely confined to the cities. Their habits before puberty are all that nature requires—plenty of pure fresh air, bodily exercise, as necessitated by their daily avocations; clothing loose and suspended from the shoulders; diet, plain, and simple but nutritious and plentiful. In girlhood they roam the fields and forests as boys, vieing with them in strength and surpassing them in many instances in the athletic sports; when suddenly puberty arrives, a change comes to them almost imperceptibly, and they are women, and very soon afterwards become mothers. Of the one hundred nullipara which the author had attended in labor and noted the age, seventy-five of them were eighteen years of age and under, the youngest being thirteen years and two months. He had heard of a case a little over eleven years of age. They were all normal labors and made good recoveris.

Owing to the lack of time, several papers were read by title and referred to the Committee on Publication.

The following officers were elected:

President—Dr. Frank M. Ridley, of LaGrange.

First Vice-President—Dr. W. H. Doughty, of Augusta.

Second Vice-President—Dr. M. L. Boyd, of Savannah.

Secretary—Dr. R. H. Taylor, of Griffin.

Treasurer—Dr. E. C. Goodrich, of Augusta.

On motion, the association adjourned to meet in Augusta, the third Wednesday in April, 1896.

After adjournment, the association enjoyed a steamboat excursion down the Savannah River to the Atlantic ocean, and partook of an "oyster roast" on Wilmington Island.

---

NOSE BLEED.—Lermoyes advises (*Med. and Surg. Rep.*) in slight cases of nose-bleed compressing the nose between the thumb and forefinger for ten minutes; if that be insufficient then apply locally a tampon moistened with a 10 per cent. solution of antipyrine, which is an excellent hæmostatic and much superior to cocaine 1.5, which latter not only has the disadvantage of being toxic, but also of being possibly followed by further hemorrhage after the vaso-constrictor action has passed away. It is also to be preferred to solutions of iron chloride, which are strong irritants and may give rise to gangrenous ulcers. In more severe cases a nasal speculum is introduced and the anterior portion of the nose tamponed with fine strips of iodoform gauze four inches in length and one in breadth. These are introduced with fine forceps. As the hemorrhages nearly always arise from the anterior portion of the nasal cavity there is no necessity of tamponing far back. Tamponade of the posterior nares is not only entirely unnecessary, but often brutally dangerous.

